# Device life of the Provox Vega voice prosthesis

**DOI:** 10.1007/s00405-012-2154-9

**Published:** 2012-09-01

**Authors:** Kelli L. Hancock, Nadine R. Lawson, Elizabeth C. Ward

**Affiliations:** 1 Speech Pathology Department, Princess Alexandra Hospital, Brisbane, QLD 4102 Australia; 2 Centre for Functioning and Health Research, Queensland Health and School of Health and Rehabilitation Sciences, The University of Queensland, Brisbane, QLD 4072 Australia

**Keywords:** Tracheoesophageal speech, Laryngectomy, Provox Vega, Device life, Indwelling voice prosthesis

## Abstract

Device life of the Provox Vega Indwelling voice prosthesis is as yet untested outside Europe. The current study examined device life and reasons for replacement within an Australian clinical setting. Twenty-three participants were monitored for device life and reasons for replacement. Main outcome measure was days to failure of initial device. Average device life and reasons for replacement were secondary measures. Initial device life data revealed 67 % had functioning devices at 3 months, 52 % at 6 months and 29 % at 12 months. Average device life was 207 days (median of 222). The majority of devices (97 %) failed due to leakage through the prosthesis. The Provox Vega Indwelling voice prosthesis had favourable device life in this cohort of patients and in comparison to European data. Reasons for replacement were consistent with international literature.

## Introduction

As in most international health services, tracheoesophageal (TE) speech is the most common method of voice rehabilitation in Australian clinical settings [1]. Advantages of TE speech over other available methods of alaryngeal speech include a more natural sounding voice, superior voice quality, improved success rates, and more immediate voice rehabilitation [1–6]. One of the few limitations of TE speech is the need for ongoing replacement of the voice prosthesis by either the clinician (for indwelling devices) or the patient (for non-indwelling devices). The most common reason for replacement of an indwelling device is leakage through the device. Other reasons for device removal include leakage around the device, or fistula-related problems including granulation tissue formation or infection [6–12].

Studies to date have revealed that, on average, the device life of an indwelling voice prosthesis falls somewhere between 4 and 6 months for the majority of patients [6, 9–16]. However, significant variation in device life has been reported within patients, between different patient groups, and across device types and geographical regions studied [17, 18]. Studies of the Provox 1 (22.5Fr) Indwelling voice prosthesis reported average device life between 102 and 311 days [10, 13–15, 19]. The Provox 2 (22.5Fr) Indwelling voice prosthesis has been reported as having an average device life between 111 and 163 days [6, 9, 12, 16]. Similar ranges have been observed across studies of the Blom–Singer Classic (20Fr) Indwelling voice prosthesis, with average device life ranging from 105 to 185 days [8, 11, 14].

Reasons proposed for the diversity in device life duration observed between studies include patient characteristics (e.g. dietary patterns, use of antifungal treatment, cleaning, controlled supraesophageal reflux), treatment characteristics (e.g. prior radiotherapy, follow-up support), as well as socioeconomic and reimbursement factors [11–13, 17, 20–23]. Another potential factor is device design. As reduced device life has negative personal and economic implications for both patient and the health service, developers have introduced a number of specialized indwelling voice prostheses. Research has shown modifications to the standard indwelling voice prosthesis design has significantly extended device life when compared to similar devices [24, 25].

Recently a new standard silicon indwelling device, the Provox Vega Indwelling voice prosthesis was introduced to the market. The only report of device life of the Provox Vega found comparable device life to the Provox 2 [17]. As both the Provox 2 and Provox Vega are constructed from silicone rubber, the results supported deterioration of the new device was comparable to other silicone devices within the same clinical setting. A second European study conducted by Lorenz and Maier [18] reported a much lower median device life for the Provox Vega. However, the authors cautioned that the median device life of 88 days (calculated from 31 devices) observed in their investigation may have been influenced by selection bias within the study sample and insufficient study duration [18]. This study included 19 participants who presented to the clinic with difficulties during a 9-month period. The authors stated that a randomized trial of 1.5–2 years duration would be more suitable to study device life to allow longer observation of device life and overall representation of both frequent and less frequent patients.

Device life is known to vary across geographical locations explained by a number of multifactoral reasons including diet, and clinical reimbursement situations [19, 20, 26]. It is therefore important to understand the expected device life of any new type of voice prosthesis within the geographical context in which it is to be used. This information can be used to inform patient choice and clinical service management. The current prospective clinical trial was designed to investigate the long-term performance of the Provox Vega Indwelling voice prosthesis in laryngectomized patients in Australia. As no prior device life studies have been published pertaining to the Australian context, a null hypothesis was assumed; that the new Provox Vega would deliver comparable device life to that reported elsewhere.

## Methods

### Ethical considerations

This prospective study was approved by the Human Research Ethics Committee of the Princess Alexandra Hospital, Queensland, Australia. It was completed under a Clinical Trial Notification (CTN) scheme and as such complied with the strict regulatory processes, including independent data analysis by both the research group and the study sponsors (Atos Medical). All participants provided informed consent prior to participation.

### Participants and procedures

This study arose from a larger randomized controlled cross-over design clinical trial that explored patient’s perceptions of two indwelling voice prosthesis systems: the Provox Vega Indwelling voice prosthesis (Atos Medical) and the Blom–Singer Classic Indwelling voice prosthesis (Inhealth) as reported elsewhere [27, 28]. These participants were recruited from the speech pathology outpatient files of the Princess Alexandra Hospital, Queensland, Australia. The inclusion criteria of that study required all participants to be TE speakers who were more than 3 months post-surgery, who had no current tracheoesophageal puncture (TEP) problems (such as enlarged TEP or infection around the TEP) or known recurrent disease at time of recruitment.

**Fig. 1:**
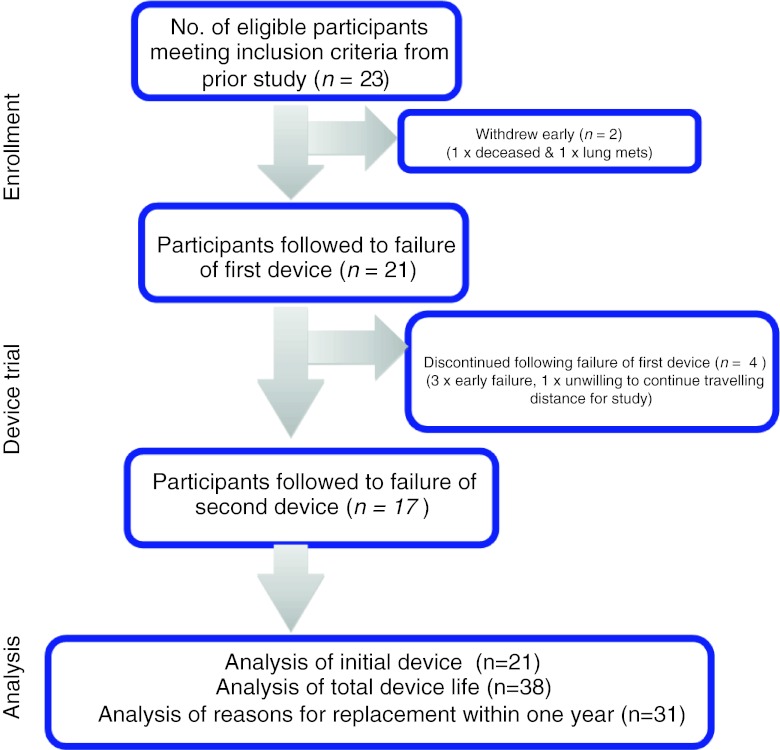
Details and justification for patient numbers and attrition throughout study

In the original study [27, 28] participants trialed both devices for a 3-week period with the order of device trial randomly allocated. The short-term component was intended to gain patient perceptions of voice effort and quality, insertion and care of devices as well as perceptual judgment of voice quality by both participants and listeners. At the termination of the above study, individuals were provided with the prosthesis of their choice. Twenty-three participants elected to continue with the Provox Vega Indwelling voice prosthesis and where possible a maximum of two devices were followed in a prospective manner until device failure. Of this current cohort of 23, two participants failed to complete the initial device trial; one deceased during the trial due to reasons unrelated to the current study or its design and one withdrew following diagnosis of further disease leaving data for 21 participants to initial device failure. Seventeen participants elected to trial a second device (Fig. 1). Four elected not to continue with the major reason being early failure of the device. For two individuals, a trial device was removed prior to device failure due to the presence of an infection in one patient and a request for early device change due to upcoming travel for the second. The data from these devices was censored. These individuals were provided a third device which was followed to true device failure and used in the study analysis as their second device.

Demographics of this study include 21 participants (17 males, 4 females, mean age = 60.19 years with age range 34–72 years). Thirteen participants had undergone a standard total laryngectomy and eight a pharyngolaryngectomy with free jejunal graft interposition. The average duration post-surgery was 4.12 years (SD = 2.5, range 0.63–10.43 years). The majority were long-term TE speech users with a mean duration using TE speech of 4.04 years (SD = 2.49, range 0.62–10.43 years). Just over half 12/21 (57 %) used 20Fr devices, while 9/21 (43 %) used 17Fr devices. The majority 18/21 (86 %) used some form of routine antifungal medication and 14/21 (67 %) reported routine use of anti-reflux medication.

At the commencement of the study, participants were instructed on cleaning and care requirements and equipment to use as per the instructions for use. Antifungal and anti-reflux medications were continued if participants were using these prior to the study. Routine clinical reviews occurred face to face or via telephone at regular intervals post-insertion of a device until device failure. Duration to device failure was recorded in days and the reasons for replacement documented. All participants with a functioning device were reviewed face to face at 12 months post-insertion following recommendations of the study device manual. The manual advised that laboratory testing of simulated usage (in the absence of bacteria and yeasts) had been performed for a period of 12 months, and as the device had not been tested beyond 12 months, usage beyond this point was at the sole discretion of the prescriber. Considering this information, all participants with a functioning device as reviewed by the treating clinician at 12 months (*n* = 6) were given the option to have the device changed. Of these, 2 participants requested a change of their initial device. The remaining participants elected not to change their device and to continue until actual device failure. For all 7 devices (across 6 participants), duration of device life data only until 365 days is used in the analysis. The actual device life duration (>365 days) of the subset of 4 individuals (across 5 devices) who continued beyond 12 months is discussed separately within the results section below.

### Statistical analyses

Kaplan–Meier survival curves were created for the patients’ initial (*n* = 21) and second (*n* = 17) Provox Vega. For this analysis, devices replaced routinely at 1 year and devices lasting beyond 1 year were all truncated at 365 days. A log-rank test (Mantel–Cox) was used to compare the device life time of the initial and the second Provox Vega. Reasons for replacement were detailed descriptively. For all analyses, significance was set at *p* < 0.05.

## Results

Early leakage before 2 months (60 days) was seen in 19 % (4/21) of the initial Provox Vega devices. The proportion of patients with a functioning device at 4, 6, 8 and 12 months was 67, 52, 43 and 29 %, respectively.

**Fig. 2:**
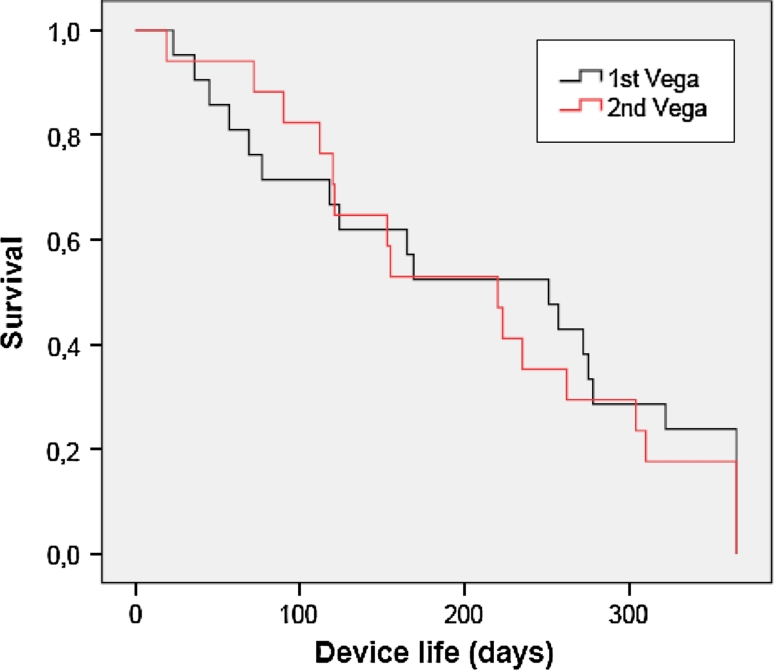
Kaplan–Meier survival curves of the patients’ initial (*n* = 21) and second (*n* = 17) Provox Vega

Figure 2 shows the plot of the survival times separated for a patients’ initial (*n* = 21) and second (*n* = 17) Provox Vega device. For this analysis, device life which extended beyond 1 year was truncated to 365 days as some devices were changed as part of routine care (as requested by participant) at 1 year. No censored devices are included in this plot. The average device life of the initial placed Vega was 208 days (SD ± 126.9, min–max: 23–365) with a median of 251 days. The device life of the second placed Vega was 205 days (SD ± 110.6, min–max 19–365) with a median of 220 days. A log-rank test comparing the survival plots for both devices revealed no significant difference (*χ* ^2^ = 0.133, *p* = 0.716) in average device life between the patients initial and second Provox Vega. The average for the total 38 Provox Vega Indwelling devices was 207 days (SD ± 118) with a median of 222 days.

Average device life was examined between the 14 participants on anti-reflux medication and the 7 who were not. Calculated using the average device life per individual, and using only device life durations up to a maximum of 365 days, the average device life of the 14 participants on anti-reflux medication was 193 days (SD = 117) and the group not on medication was 206 days (SD = 120). There was no significant (*t* = −0.252, *p* = 0.804) difference between the groups.

For the 4 participants (2 on anti-reflux medication, 2 not) who continued with their initial or second device beyond 12 months, 3 had one device last longer than 1 year (398, 693, 486 days) and 1 had 2 devices last longer than 1 year (initial device = 383 days, second device = 445 days).

Thirty-one devices were replaced within 1 year (Table 1). The majority of those devices 30/31 (97 %) failed due to leakage through the prosthesis and the one remaining device (3 %) failed due to patient report of slight changes in voice effort and voice quality. Nil incidents of leakage around the voice prosthesis were observed in this cohort. Five devices remained in situ for longer than 1 year. Three of these were removed for device failure, two, because of leakage through the voice prostheses at 398 and 383 days, and one for noted changes in voice effort at 486 days. The remaining 2/5 devices were replaced at 445 and 693 days for management of granulation formation and long time device in situ, respectively.

**Table 1:** Summary Information regarding sizes and reasons for replacement of initial and second Vega within 12 month mark (*n* = 31)

Device	Outer diameter	Length	Reasons for replacement (*n* = 31)
First Vega (*n* = 17)	20 Fr: 11	4 mm: 1	Leakage through: 16 (52 %)
	17 Fr: 6	6 mm: 1	
		8 mm: 7	Increased speaking effort: 1 (3 %)^a^
		10 mm: 8	
Second Vega (*n* = 14)	20 Fr: 8	6 mm: 2	Leakage through: 14 (45 %)
	17 Fr: 6	8 mm: 3	
		10 mm: 7	
		12.5 mm: 2	

^a^17Fr 8 mm Provox Vega

## Discussion

For the initial Provox Vega device trial (*n* = 21), early failure was observed in only a small proportion of the group (19 %.). This is consistent with previous literature which has reported up to 20 % can experience early leakage within the first month [8, 16]. Examination of pre trial data revealed a history of early failure with other devices for these participants. At 6 months more than half the group (52 %) continued with their initial device, while at 12 months, 29 % continued with their original device. Laccourreye et al. [10] reported from a cohort of 100 devices that 65.9 and 23.7 % were in situ at 6 and 12 months post-insertion, respectively. These results suggest for this cohort the Provox Vega is a suitable voice prosthesis with favourable device life in those that do not have a current history of early device failure.

The reasons for device failure and change in this study were primarily leakage through the device (97 %), consistent with previous literature [7, 9, 17, 19]. There were no incidences of leakage around or other significant complications as the reason for replacement. This may be related to the fact that the trial excluded patients with current TEP problems. One finding of note was the instance for one participant where device failure was identified by noted changes to voice quality and an obvious increase in speaking effort both within and outside the 12-month timeframe (278 and 486 days). Clinical examination confirmed no leakage, and eliminated TEP causes for voice changes and together with improved voice on replacement suggest a change to the opening/closing functionality of the valve mechanism within the prosthesis.

The average device duration for the 38 devices of 207 days (median 222) was found to exceed other studies on device life for the Provox Vega (i.e. median device life of 74–93 days). In comparison average device life was almost 3 months longer for this cohort. Hence the null hypothesis set for this study is rejected. Comparison against other standard silicon indwelling devices illustrated device life is comparable to studies of the Provox 1 device [14, 15] and exceeds those for the Provox 2 or Blom–Singer Classic Indwelling Voice Prostheses [6, 9, 12, 16]. To date there have been no other studies of indwelling device life within the Australian context. It is unclear whether longer device life is a potential characteristic of this geographical region or the device itself. Possible factors which may account for this increase in device life could relate to increased patient monitoring during the study period and patterns of cleaning and care. The rigorous requirements of this clinical trial ensured patients were followed regularly and monitored between insertion and replacement of the device. Certainly some studies have identified a positive influence on device life with increased patient follow-up [22]. Participants were instructed to care and clean their Provox Vega voice prosthesis as per the instructions for use. The provision of appropriate cleaning equipment (brushes and flushes) and compliance with recommendations were regularly discussed at face to face or phone review sessions. Prior to the study, flushes were not used in the cleaning routines of any patients and cleaning and care was not regularly discussed in sessions beyond the initial training and skills development sessions or as clinically warranted. Involvement in the larger clinical trial (from which the current cohort was recruited) identified a patient preference for the cleaning equipment of the Provox Vega (brush and flush) when compared to a comparator device in a short-term trial [28]. Hence the current participants may have had greater compliance to cleaning routines than prior to the trial. Free et al. [20] explored the relationship of regular airflow provided via the Provox Flush cleaning device on biofilm development and found the flush had a cleansing effect, reducing biofilm formation which could potentially assist with device life. It is possible that using both a brush and a flush and improved patient compliance with cleaning may have influenced device life in the current cohort. This issue warrants further investigation.

Boscolo-Rizzo et al. [11] suggested a possible association between uncontrolled reflux and limited device life, with the mean device life of patients with reflux being just over half of those without. Lorenz et al. also demonstrated that the lifespan of the voice prosthesis is reduced in those with reflux symptoms [18]. Considering that over half of the current patient cohort who reported experiencing reflux were taking regular anti-reflux medications to control their symptoms, it is possible this may be a favourable influencing factor on device life. Whilst in our study the device life of participants who were receiving and not receiving anti-reflux medication was not found to be significantly different, the potential impact of uncontrolled reflux on device life remains a possible factor which should be considered for patients experiencing limited device life. In addition to reflux medication, the majority of participants (18/21, 86 %) from this cohort were also regular users of antifungal medications prior to this study, and continued their usual practice during the Provox Vega trial. This may have had an impact on extended device life and warrants further investigation.

One final factor relates to individual patient behaviour, and their tolerance of periods of minor leakage prior to requesting device change. Within Australia and other international settings there are issues relating to purchasing and provision of equipment and its reimbursement. Clinicians and patients are acutely aware of the costs associated with short device life where funding restrictions apply for voice prostheses. Furthermore, within Australia the geographical expanse of the country and distances necessary to travel for device replacement may foster increased patient tolerance of mild intermittent leakage prior to prosthesis replacement. Cornu et al. [13] described this behaviour/phenomenon as patients taking “a more conservative approach” and proposed that it may have contributed to the extended device life in their cohort in South Africa. While the potential for this phenomenon to have influenced device life in the current study cannot be fully discounted, the increased monitoring of patients in the study largely prevented this from occurring and replacements were identified promptly and device changes actioned in a timely manner. In future studies, informing patients to contact the study team at the point of *initial* identification of any leakage would help ensure this possible source of data bias was controlled for.

Examination of the individual device durations highlighted considerable variability both within individuals and between participants in the current group. Within this clinical setting, the authors have observed variation in device life of individual patients as a regular occurrence, regardless of types of devices (indwelling or non-indwelling or different manufacturers). Other researchers have also noted high intra-individual differences in device life [18]. There does not appear to be one clear reason to explain this variability. It is more likely the result of a multitude of factors that may not be controlled for (e.g. fluctuations in patient health; dietary changes; climate changes, etc.). Acknowledging this degree of inherent variability in device life of indwelling prostheses exists it may be optimal to trial at least two devices prior to determining suitability of a device and potential device life for any individual.

## Conclusion

Results demonstrate that for this clinical cohort, the Provox Vega Indwelling voice prosthesis was a favourable device in the Australian context with an average device life of 207 days (median 222). Reasons for device life failure were consistent with prior literature. In Australia equipment provision for this clinical population is subject to financial pressures and as a consequence device life is an important factor when selecting an appropriate indwelling device for both patients and clinicians. Further exploration of the possible relationship between cleaning practices, equipment and device life is warranted.
